# Mitochondrial dysfunction by glyoxalase 1 deficiency disrupts definitive endoderm and alveolar development of human pluripotent stem cells

**DOI:** 10.1038/s12276-025-01524-y

**Published:** 2025-09-01

**Authors:** Suji Jeong, Hyebin Koh, Minje Kang, Ji-Young Kim, Roya Rasaei, Woo Jin Kim, Seon-Sook Han, In Sun Hong, Se-Ran Yang, Jong-Hee Lee, Seok-Ho Hong

**Affiliations:** 1 Department of Internal Medicine, School of Medicine, Kangwon National University, Chuncheon, Republic of Korea; 2 National Primate Research Center, Korea Research Institute of Bioscience and Biotechnology, Cheongju, Republic of Korea; 3 Department of Advance Bioconvergence, KRIBB School of Bioscience, University of Science and Technology, Daejeon, Republic of Korea; 4 Department of Internal Medicine, Kangwon National University Hospital, Chuncheon, Republic of Korea; 5 Department of Biochemistry, School of Medicine, Gachon University, Incheon, Republic of Korea; 6 Department of Thoracic and Cardiovascular Surgery, School of Medicine, Kangwon National University, Chuncheon, Republic of Korea; 7 KW-Bio, Chuncheon, Republic of Korea

**Keywords:** Stem-cell differentiation, Induced pluripotent stem cells

## Abstract

Normal mitochondrial function is essential for human induced pluripotent stem (hiPS) cell differentiation into definitive endoderm (DE). However, the underlying mechanisms that maintain mitochondrial homeostasis during DE differentiation are not fully elucidated. Here we report that glyoxalase 1 (GLO1) is a novel regulator of DE differentiation and subsequent alveolar development in hiPS cells via maintaining mitochondrial homeostasis. To determine the role of GLO1 in these processes, we first established GLO1-knockout hiPS cells using CRISPR–Cas9-mediated genome deletion and demonstrated that GLO1 deficiency significantly reduced the differentiation efficiency of DE, leading to defects in alveolar epithelial cell differentiation and alveolar organoid development. Moreover, GLO1 deficiency interfered with mitochondrial biogenesis and respiration during the early DE stage. Defects in DE differentiation due to dysfunctional mitochondria were effectively rescued by high-dose treatment with CHIR99021, a glycogen synthase kinase 3 inhibitor. Our study uncovered an essential role of GLO1 as a key regulator of mitochondrial homeostasis for early lineage specification of hiPS cells, moving away from its conventional role as a primary enzyme in methylglyoxal detoxification.

## Mitochondrial function linked to GLO1 in stem cells

The study explores how a protein called glyoxalase 1 (GLO1) affects the development of lung cells from human induced pluripotent stem (hiPS) cells. Researchers found that GLO1 is crucial for the proper formation of definitive endoderm (DE). They used CRISPR–Cas9, a gene-editing tool, to create hiPS cells without GLO1 and observed that these cells struggled to develop into DE and lung cells. This was linked to problems with mitochondria, the cell’s energy producers. The team tested if a chemical called CHIR99021 could fix these issues. CHIR99021 is known to help cells develop by activating a pathway involving β-catenin, a protein important for cell growth. High doses of CHIR99021 improved mitochondrial function and helped the GLO1-deficient cells develop properly. The study concludes that GLO1 plays an essential role in early cell development by maintaining healthy mitochondria.

This summary was initially drafted using artificial intelligence, then revised and fact-checked by the author.

## Introduction

Definitive endoderm (DE) specification is a critical step in the development of various endoderm germ layer-derived organs^1,2^. In the context of lung development, DE specification sets the stage for the subsequent differentiation of lung epithelial cells and the formation of complex alveolar structures^1,2^. Impairment of this process can have a significant impact on lung development, leading to birth defects, respiratory diseases and later respiratory dysfunction. Therefore, understanding the precise signaling mechanisms that govern DE specification is crucial to comprehending the complexities of lung development and devising strategies to generate functional alveolar epithelial cells (AECs) from human pluripotent stem (hPS) cells.

Exit from the pluripotent state and priming for differentiation into specific lineages of hPS cells is accompanied by a metabolic switch from glycolysis toward oxidative phosphorylation (OXPHOS)^3–5^. Mitochondria play a pivotal role in this metabolic remodeling, as they are the core organelles of OXPHOS and are the essential power plants for generating the energy needed for hPS cell differentiation^6,7^. Recent studies have demonstrated the significance of mitochondrial homeostasis in the differentiation of hPS cells into the DE lineage. Qi et al. revealed that transcription factor A, mitochondrial (TFAM) deficiency compromises mitochondrial structure, impairs cell proliferation by inducing cell-cycle arrest and hinders the self-renewal capacity of hPS cells. This depletion also curtailed the ability of the cells to differentiate into the DE germ layer^8^. Lv et al. demonstrated that TFAM is necessary to maintain normal mitochondrial homeostasis, which is regulated by adenosine triphosphate (ATP) and reactive oxygen species for the proper DE specification of hPS cells^9^. In addition, transforming growth factor (TGF)-β-dependent mitochondrial biogenesis is activated during the final endodermal differentiation process^10^. However, the upstream signals and molecules that regulate mitochondrial homeostasis during the DE differentiation process remain incompletely understood.

Glyoxalase 1 (GLO1) is a key enzyme in the glyoxalase system that is primarily responsible for detoxifying methylglyoxal (MG), a reactive metabolite that forms advanced glycation end products, leading to protein dysfunction^11,12^. Beyond its conventional detoxification role, GLO1 is increasingly recognized for its involvement in regulating mitochondrial homeostasis, which is closely associated with the development of metabolic and psychiatric disorders^13–17^. In addition, one study reported that a decreased level of GLO1 leads to long-lasting alterations in adult neurons and neural precursor cell pools postnatally^18^. Although mitochondrial dysfunction related to this abnormal neurogenesis has not been investigated in this study, it strongly suggests a critical role of GLO1 in early embryonic development. Thus, herein, we asked if GLO1 is essential for DE specification and subsequent AEC differentiation via regulating mitochondrial homeostasis.

To verify our conjecture, we first generated a GLO1-knockout (GLO1^−/−^) human induced pluripotent stem (hiPS) cell line and compared its early DE specification and late alveolar organoid (AO) developmental capacity with those of a wild-type (WT) control. We also checked the mitochondrial morphology and activity during DE specification to determine the potential role of GLO1 in mitochondrial homeostasis. Then, we analyzed whether defects in mitochondrial function and DE specification due to GLO1 deficiency can be recovered by modulating DE-related signaling using pharmacological inhibitors.

## Materials and methods

### Maintenance of hiPS cells

In brief, WT hiPS cells (BM-iPSC C3)^19^ and GLO1^−/−^ hiPS cells were cultured under xeno- and feeder-free conditions using TeSR-E8 (STEMCELL Technologies, #05990) on dishes coated with Matrigel^T^ (Corning, #356231). At 80% confluency, cells were passaged every 4–5 days by ReLeSR (STEMCELL Technologies, #05872).

### Generation of the GLO1-knockout hiPS cells by CRISPR–Cas9 genome editing

The targeting vector designed for knockout of *GLO1* expression was cloned into a T-easy vector, which is modified to contain a floxed Geneticin resistance gene (Neo) under the PGK promoter. The PGK-Geneticin-*GLO1* knockout donor plasmid contains left (a 1.4-kb fragment, spanning upstream of the *GLO1* gene including initiation codon) and right (a 1.5-kb fragment, containing *GLO1* intron) homology arms. The genomic sequences of *GLO1* were obtained from the National Center for Biotechnology Information (NCBI) database (Ensembl: ENSG00000124767), and each homology arm was amplified from the genomic DNA of hPS cells. The *GLO1*-knockout episomal clustered regularly interspaced short palindromic repeats (CRISPR)–Cas9 plasmid contains *GLO1* single guide RNA to target the sequence (GTCGGAGCAGCAACTGAGGG). Next, hiPS cells at 70–80% confluence were collected with Accutase (Sigma-Aldrich), and 5 × 10^5^ single cells were resuspended in 100 μg *GLO1*-knockout plasmid and 1.5 μg spCas9 *GLO1* guide RNA (1.5 μg) and then transferred to a single nucleocuvette. Nucleofection was performed with 4D-Nucleofector X Kit L (Lonza) following the manufacturer’s instructions immediately after electroporation. Cells were transferred into one well of a Vitronectin XF or Matrigel-coated six-well cell culture plate (Corning) containing 1 ml of TeSR-E8 medium with 5 μM ROCK inhibitor (Y-27632, Medchem Express). Medium was changed every 24 h, and 500 ng/ml Geneticin (Gibco) was added for the selection of transfected cells 48 h after genome editing. Single colonies grown sufficiently were transferred into one well of a 24-well cell culture plate and further analyzed by quantitative PCR (qPCR) and western blotting to confirm genome editing.

### RNA isolation and real-time qPCR

Total RNA was isolated from hiPS cells, DE cells, alveolar epithelial progenitors (AEPs), AECs and lung organoid (LO) using the RNeasy Mini Kit (Qiagen, #74004), and cDNA was synthesized using TOPscrip RT DryMIX (Enzynomics, #RT200). PCR amplification was performed using a Step One Plus real-time PCR system (Applied Biosystems) with TOPreal qPCR 2X PreMIX (Enzynomics). All the mRNA expression was normalized to GAPDH. The primer sequences for human genes are listed in Supplementary Table 1.

### Stepwise differentiation of hiPS cell-derived AECs

A stepwise direct AEC induction was performed as previously described^20^. In brief, undifferentiated hiPS cell colonies were seeded at a low density, with fewer than ten colonies per well. When the colonies grew to approximately 1 mm in diameter, AEC differentiation was initiated with exposure to a sequential induction medium.

### Generation of AOs from hiPS cells and measurement of AO diameter

AOs were generated as previously described^20^. In brief, two-dimensional cultures were dissociated on day 21 of AEC differentiation and seeded into 96-well round-bottom plates containing AEC maturation medium supplemented with 10 μM ROCK inhibitor (STEMCELL Technologies). Plates were centrifuged at 450*g* for 10 min and incubated overnight at 37 °C in a humidified atmosphere containing 5% CO_2_. After overnight culture, the aggregates were transferred to six-well low-attachment plates (Corning) containing fresh AEC maturation medium and cultured for 7 days to generate AOs. Subsequently, bright-field microscopy images of the AOs on day 28 were measured for length and width using the ImageJ program. The measured value was calculated by applying a method for measuring the size of spherical fat cells, as follows.The radius of the AOs (*r*), defined as the average of length and width, was calculated from measurements obtained using ImageJ. Assuming a spherical shape for the AOs, the diameter was estimated using the formula $A=\pi {r}^{2}$ (refs. ^21,22^).

### Stepwise generation of anterior foregut (AF) spheroids and LOs

A stepwise generation of AF spheroids and LOs from hiPS cells was performed as previously described^23^. In brief, AF spheroids begin to form around day 3–4 of AFE differentiation and lift away from the monolayer to float in the induction medium. These AF spheroids can generally be observed from days 6 to 12. Floating AF spheroids were collected and then cultured in a Matrigel droplet to direct into LOs for up to 27 days.

### Flow cytometric analysis

The hiPS cells were collected between days 0, 4, 14 and 21 of AEC differentiation. Adherent cells were incubated with cell dissociation buffer (Gibco, #13151014) for 10 min. Cells passed through a 70-µm cell strainer were incubated with the following fluorochrome-conjugated anti-human antibody at 4 °C for 1 h. Nuclear markers were fixed and permeabilized using the Cytofix/Cytoperm Fixation/Permeabilization Kit (BD Pharmingen, #554714), which contains 4.2% formaldehyde, for 20 min at room temperature, followed by additional steps according to the manufacturer’s instructions. A corresponding isotype control antibody was used for each primary antibody to set up the negative control, and detailed information on human antibodies is available in Supplementary Table 2. Dead cells were excluded by staining with 7-amino actinomycin D. Frequencies were measured using a FACS Canto TM II and Accuri C6 flow cytometers (BD Biosciences), and the acquired data were analyzed with FlowJo software (Tree Star).

### Annexin V-FITC/PI staining

Externalization of phosphatidylserine was determined by flow cytometry of cell staining using Annexin V-FITC conjugate and PI according to the kit manufacturer’s protocol. The cells were collected with cell dissociation buffer, washed with phosphate-buffered saline (PBS), centrifuged at 450*g* for 5 min to discard the supernatant and resuspended in 100 μl binding buffer (FITC Annexin V Apoptosis Detection Kit with propidium iodide (PI), BioLegend, #640914). Subsequently, Annexin V-FITC (5 μl) and PI (5 μl) were added to the suspension and incubated for 15 min at room temperature in the dark. The cells were evaluated by flow cytometry using FACS Canto II and an Accuri C6 flow cytometers (BD Biosciences). Data were analyzed using FlowJo software (Tree Star), and the results are expressed as percentages of the cell populations.

### Immunocytochemistry staining

Cells after 4 days of differentiation were fixed with 4% paraformaldehyde for 20 min at room temperature. The cells were blocked by 5% bovine serum albumin (Sigma, #9048-46-8) in PBS for 1 h at room temperature and permeabilization with 0.1% Triton X-100 for 10 min at room temperature. The samples were rinsed with 0.03% Triton X-100 and subsequently probed with primary antibodies against OCT4 (Abcam, #ab200834, 50 μg/ml), SOX17 (Abcam, #ab224637, 0.2 μg/ml), CXCR4 (Abcam, #ab124824, 50 μg/ml) and active β-catenin (Cell Signaling, #8814, 1:1,000) in 5% bovine serum albumin in PBS for 1 h at room temperature. As the next step, cells were followed by incubation with secondary antibodies for 1 h at room temperature. The cells were counterstained using Fluoroshield with a DAPI histology mounting medium (Abcam, #104139). Immunocytochemistry staining images were captured under fluorescence microscopy.

### Western blot analysis

Protein extracted from undifferentiated hiPS cells and differentiated cells were lysed in protein lysis buffer and quantified using the bicinchoninic acid protein assay. The 20 μg of sample protein were separated by SDS–PAGE (10%) gel and then transferred to polyvinylidene fluoride membranes. Nonspecific binding proteins were blocked with 5% skim milk for 1 h at room temperature. The membranes were incubated with primary antibodies against GLO1 (Abcam, #ab96032, 1:1,000), active β-catenin (Cell Signaling, #8814, 1:1,000) and MG-H1 (Cell Biolabs, #STA-011, 1:1,000) overnight at 4 °C. The chemiluminescence signal was scanned with a ChemiDOC imaging system (Bio-Rad Laboratories).

### Transmission electron microscopy

Specimens were fixed with 2% glutaraldehyde and 2% paraformaldehyde in 0.1 M phosphate buffer (pH 7.4) for 24 h at 4 °C and washed in 0.1 M phosphate buffer, post-fixed with 1% OsO4 in 0.1 M phosphate buffer for 1.5 h. Then, the gradually increasing concentrations of ethanol (50–100%) were used for dehydration. Specimens were infiltrated with propylene oxide for 10 min and embedded with a Poly/Bed 812 kit (Polysciences, #08792-1), polymerized at 60 °C for 18 h. The specimens were sectioned (200 nm) with a diamond knife in the ultramicrotome (EM-UCT, Leica) and stained with toluidine blue for observation by an optical microscope. Thin sections (70 nm) were double-stained with 5% uranyl acetate for 10 min and 1% lead citrate for 5 min. The specimens were observed in a field-emission transmission electron microscope (JEM-1011, JEOL) at an acceleration voltage of 80 kV and photographed with a digital charge-coupled device camera (RADIUS, EMSIS).

### MitoTracker staining and live-cell imaging

Mitochondrial mass was detected for live-cell imaging by incubating with 25 nM MitoTracker Green FM (Thermo Fisher Scientific, #M7514) for 30 min at 37 °C. Immunostaining and live-cell images were captured using a K1-Fluo (Nanoscope Systems) confocal microscope. The mean fluorescence intensity (MFI) was analyzed with ImageJ software.

### Mitochondrial respiration and glycolytic assay

Oxygen consumption rate (OCR) and extracellular acidification rate (ECAR) were measured using the Seahorse XF HS mini Bioanalyzer (Seahorse Bioscience) as previously described^24^. OCR and ECAR measurements were performed according to the manufacturer’s protocol. In brief, cells were seeded at a concentration of 15,000 cells per well on XFp Cell Culture Miniplates 48 h before the start of measurement. To measure OCR, the concentrations of oligomycin, FCCP and rotenone/antimycin A (R/A) were sequentially injected at 3, 1 and 1 μM, respectively. For ECAR measurements, concentrations of R/A and 2-deoxy-glucose were sequentially injected at 1 μM and 50 mM, respectively. The results of the Seahorse XF assays were normalized to the total cell number.

### ATP measurements

ATP rate was measured using the Seahorse XF HS Mini Bioanalyzer (Seahorse Bioscience) as previously described^24^. ATP measurements were performed according to the manufacturer’s protocol. In brief, cells were seeded at a concentration of 15,000 cells per well on XFp Cell Culture Miniplates 48 h before the start of measurement. To measure ATP levels, oligomycin and R/A were sequentially injected at concentrations of 3 μM and 1 μM, respectively. The results of the Seahorse XF assays were normalized to the total cell number.

### Statistical analysis

The statistical analysis was executed using GraphPad Prism 10 (GraphPad). Values for all measurements are presented as means ± standard deviation (s.d.). Differences among the groups were evaluated using the Student’s *t*-test or one- and two-way analysis of variance (ANOVA) with the Tukey and Bonferroni multiple-comparisons tests, as appropriate. A *P* value of less than 0.05 was considered statistically significant.

## Results

### GLO1 deficiency disturbs hiPS cell-derived DE differentiation

First, we investigated the temporal expression pattern of *GLO1* during hiPS cell-derived DE differentiation and observed gradual increases in the transcription of *GLO1* along with other DE-related genes, including SRY-box transcription factor 17 (*SOX17*) and CXC motif ligand 4 (*CXCL4*) (Supplementary Fig. 1a). Western blot and quantitative reverse transcription PCR (qRT-PCR) analyses revealed a gradual increase in GLO1 protein and transcript expression, peaking at day 14, when AEPs emerged (Supplementary Fig. 1b,c). These findings suggest that GLO1 may be involved in regulating DE and subsequent AEC differentiation.

**Fig. 1:**
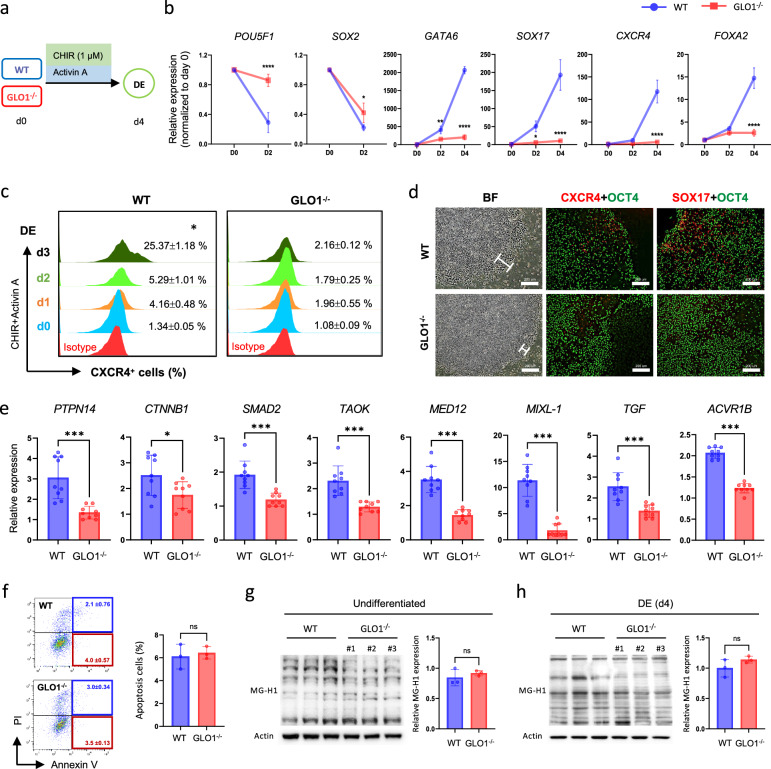
GLO1 deficiency disturbs hiPS cell-derived DE differentiation. **a** Scheme of the DE differentiation of hiPS cells. **b** qRT-PCR analysis of pluripotency (*POU5F1* and *SOX2*) and DE-specific genes (*SOX17*, *CXCR4*, *GATA6* and *FOXA2*) in WT and GLO1^−/−^ hiPS cells at day 4. Data are normalized to the mRNA levels from day 0. **c** Flow cytometric analysis to determine temporal induction efficiency between WT and GLO1^−/−^ hiPS cells. CXCR4^+^ cells represent DE cells. Isotype: an antibody that lacks specificity for the target proteins but matches the immunoglobulin class of the primary antibody. **d** Immunofluorescence staining of DE cells at day 4 of differentiation. CXCR4 (red) and SOX17 (red) served as DE markers. OCT4 served as a pluripotency marker. In the bright-field image, the white marking designates the area in which differentiation occurs, extending from the edge of the colony. Scale bars, 200 μm. **e** qRT-PCR analysis for DE-positive regulators in WT and GLO1^−/−^ hiPS cells. **f** Apoptosis analysis of WT and GLO1^−/−^ hiPS cells using Annexin V and PI staining. Apoptosis cells (%) were calculated by summing the Annexin V^+^PI^−^ (early apoptosis, red box) and Annexin V^+^PI^+^ (late apoptosis, blue box) populations, and the combined values are represented in the graph. **g** Western blot analysis of MG-H1 protein expression in undifferentiated WT and GLO1^−/−^ hiPS cells. **h** Western blot analysis of MG-H1 protein expression in WT and GLO1^−/−^ hiPS cells at DE day 4. The sum of the band densities from all detected bands was analyzed, and the resulting values are shown in the bar graph. All data are shown as the means ± s.d. *n* = 3. Statistical analysis was performed using a two-way ANOVA and Šídák’s multiple-comparisons test (**b**) or unpaired Student’s *t*-test (**c**, **d**, **f** and **g**). *P < 0.05, **P < 0.01, ***P < 0.001, ****P < 0.0001.

To further determine the biological function of GLO1 in DE specification, we generated a GLO1^−/−^ hiPS cell line using clustered regularly interspaced short palindromic repeats (CRISPR)–Cas9-mediated genome deletion (Supplementary Fig. 2a–f). We found that undifferentiated GLO1^−/−^ hiPS cells were morphologically indistinguishable from WT hiPS cells and that the expression levels of the core pluripotency markers (POU domain, class 5 transcription factor 1 (*POU5F1*), homeobox transcription factor Nanog (*NANOG*) and *SOX2*) and the undifferentiated markers (stage-specific embryonic antigen-4 (SSEA-4) and podocalyxin (TRA-1-60)) were not affected (Supplementary Fig. 2g,h). Then, we conducted side-by-side comparisons evaluating the ability of WT and GLO1^−/−^ hiPS cells to differentiate into DE using a simple and rapid DE induction protocol (Fig. 1a). Real-time qPCR analysis revealed marked reductions in the expression levels of DE-related genes, including GATA-binding factor 6 (*GATA6*), *SOX17*, *CXCL4* and forkhead box protein A2 (*FOXA2*), in GLO1^−/−^ hiPS cells at day 4 compared with the WT cells, whereas GLO1^−/−^ hiPS cells expressed higher levels of *OCT4* and *SOX2* compared with the WT cells (Fig. 1b). Similarly, flow cytometry analysis showed that GLO1^−/−^ hiPS cells exhibited a significant reduction of CXCR4^+^ DE cells at day 3 of DE differentiation (GLO1^−/−^ versus WT, 25.36 ± 1.18% versus 7.86 ± 4.41%, *P* < 0.01) (Fig. 1c and Supplementary Fig. 3). We also confirmed this finding by immunofluorescence staining of day 3 differentiated cells for CXCR4 and SOX17 (Fig. 1d). Recently, positive regulators of DE differentiation primarily associated with Hippo, Wnt and Nodal pathways have been identified using genome-scale CRISPR screens^25^. Thus, we further analyzed the expression patterns of some positive regulators, and the results showed significant decreases in the expression levels of these genes in GLO1^−/−^ hiPS cells compared with the WT cells (Fig. 1e). This reduced differentiation capacity of GLO1^−/−^ hiPS cells does not appear to be due to apoptosis, necrosis (Fig. 1f and Supplementary Fig. 4) or cytotoxicity of accumulated MG (Fig. 1g, h). These results indicate that GLO1 deficiency disturbs DE differentiation but does not affect the maintenance of the undifferentiated state of hiPS cells.

### GLO1 deficiency negatively influences lung epithelial commitment and AO development

**Fig. 2:**
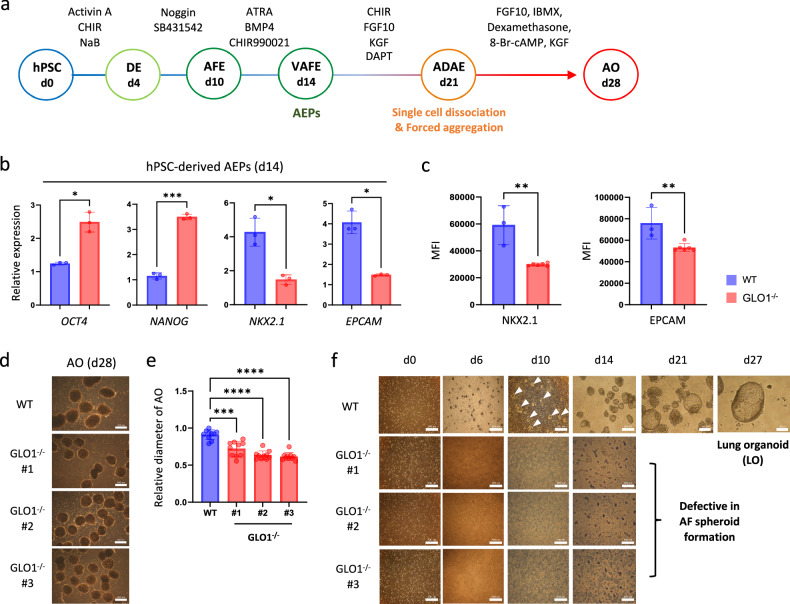
Effects of GLO1 efficiency on AEP differentiation, LO and AO development. **a** Schematic of the stepwise induction protocol for generating AECs and AOs from hiPS cells. VAFE, ventralized alveolar foregut endoderm; ADAE, airway distal alveolar epithelium. **b** Transcript levels of pluripotency (*OCT4* and *NANOG*)- and AEP (*NKX2.1* and *EPCAM*)-related genes in WT and GLO1^−/−^ hiPS cells at day 14 of differentiation. **c** MFIs of NKX2.1^+^ and EPCAM^+^ cells in WT and GLO1^−/−^ hiPS cells at day 14 of differentiation. **d** Representative bright-field images of AO generated from WT and GLO1^−/−^ hiPS cells at day 28 of differentiation. Scale bars, 200 μm. **e** AO diameter was measured on day 28 of the organoid culture, and the mean was calculated (ten AOs per group). **f** Representative bright-field images at various days of differentiation, including day 0 (24 h after single-cell plating), days 6–10 (AF endoderm, AFE) and days 14–27 (collection of floating AF spheroids and induction to LOs). AF spheroids (white arrows) begin to form on approximately days 3–4 of AFE differentiation. Scale bars, 100 and 200 μm. hPSCs, hPS cells. All data are shown as the means ± s.d. *n* = 3 (**b** and **c**) and *n* = 10 (**e**). Statistical analysis was performed using a one-way ANOVA and Dunnett’s multiple-comparisons test (**e**) or unpaired Student’s *t*-test (**b** and **c**). *P < 0.05, **P < 0.01, ***P < 0.001, ****P < 0.0001.

Next, we asked if the defects in DE specification due to GLO1 deficiency in hiPS cells affect subsequent alveolar development. To answer this question, we plated hiPS cells as colonies for direct differentiation to the alveolar lineage using the established stepwise induction protocol^20^ (Fig. 2a). qPCR analysis revealed significant reductions in AEP markers (NK2 homeobox 1 (*NXK2.1*) and epithelial cell adhesion molecule (*EPCAM*)) in GLO1^−/−^ hiPS cells at day 14 compared with the WT cells (Fig. 2b). Flow cytometry analysis also showed that the MFIs of NKX2.1 and EPCAM in GLO1^−/−^ hiPS cells were significantly lower compared with those in WT cells (Fig. 2c). Then, we formed three-dimensional forced aggregates from single-cell dissociation at day 21 to generate AOs (Fig. 2a). Interestingly, the average diameter of the resulting AOs was significantly smaller in GLO1^−/−^ AOs compared with that of WT AOs (Fig. 2d, e). These findings demonstrate that impaired DE differentiation due to GLO1 deficiency negatively influences lung epithelial commitment and AO development. We further investigated the effects of GLO1 deficiency on the late stage of alveolar development using a previously established LO protocol^23^. This protocol contains an AF spheroid formation step, which can differentiate into LOs containing AECs. We observed the formation of floating AF spheroids on approximately days 8 and 9 of WT hiPS cell culture and the subsequent development of LOs (Fig. 2f). qRT-PCR analysis revealed the robust expression of markers for distal tip lung progenitors (*GATA6* and inhibitor of DNA binding 2 (*ID2*)), type 1 AECs (aquaporin 5 (*AQP5*) and HOP homeobox (*HOPX*)), type 2 AECs (surfactant protein B (*SFTPB*) and *SFTPC*) and mesenchymal stromal cells (*VIMENTIN*) in LOs compared with undifferentiated hiPS cell cultures (Supplementary Fig. 5). Unexpectedly, under this LO protocol, GLO1^−/−^ hiPS cells did not form AF spheroids (Fig. 2f), which did not allow us to further compare LO development capacity with WT hiPS cells. However, these findings suggest that defects in DE differentiation due to GLO1 deficiency may also negatively influence subsequent AF endoderm development.

### GLO1 deficiency impairs mitochondrial homeostasis

**Fig. 3:**
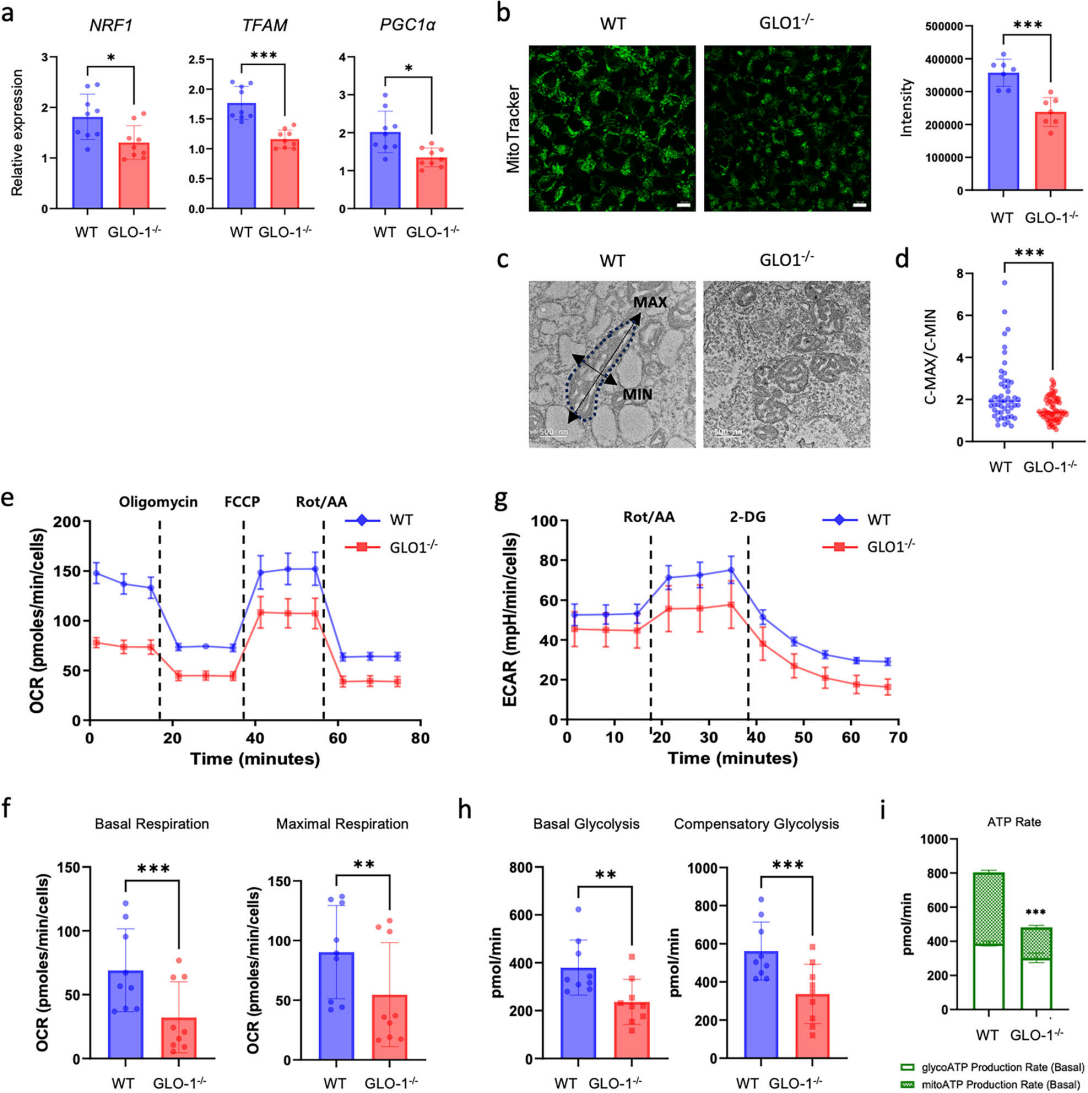
GLO1 deficiency impairs mitochondrial homeostasis in hiPS cells. **a** Relative mRNA expression levels of mitochondrial biogenesis-related genes (*NRF1*, *TFAM* and *PGC1α*) in undifferentiated WT and GLO1^−/−^ hiPS cells were determined by qRT-PCR. **b** Mitochondrial masses in WT and GLO1^−/−^ hiPS cells were measured by MitoTracker Green FM staining analysis. Scale bars, 100 μm. **c** Representative TEM images of mitochondrial structures in WT and GLO1^−/−^ hiPS cells. Scale bars, 500 nm. **d** Calculated mitochondrial C-MAX (long-axis length)/C-MIN (short-axis length) values in WT and GLO1^−/−^ hiPS cells. **e** The kinetic profile of the OCR was measured in undifferentiated WT and GLO1^−/−^ hiPS cells using the Seahorse XF Real-Time assay. Black lines show times of treatment with oligomycin, FCCP and rotenone/antimycin A (Rot/AA). **f** The basal respiration and maximal respiration in undifferentiated WT and GLO1^−/−^ hiPS cells. **g** The kinetic profile of the ECR was measured in WT and GLO1^−/−^ hiPS cells using the Seahorse XF Real-Time assay. Black lines show times of treatment with Rot/AA and 2-DG. **h** The basal and compensatory glycolysis in WT and GLO1^−/−^ hiPS cells. All data are shown as the means ± s.d. *n* = 3 (**a** and **e**–**i**), *n* = 7 (**b**), *n* = 51 (WT hiPS cells in **c**) and *n* = 69 (GLO1^–/–^ hiPS cells in **c**). Statistical analysis was performed using a two-way ANOVA (**i**) or unpaired Student’s *t*-test (**a**, **b**, **d**, **f** and **h**). *P < 0.05, **P < 0.01, ***P < 0.001.

The maintenance of normal mitochondrial homeostasis is essential for the DE specification of hiPS cells. Thus, we assumed that GLO1 might be involved in mitochondrial function during hiPS cell-derived DE specification. Indeed, mitochondria biogenesis-related genes, including nuclear respiratory factor-1 (*NRF1*), *TFAM* and peroxisome proliferator-activated receptor-γ coactivator 1-α (*PGC1α*) were downregulated in undifferentiated GLO1^−/−^ hiPS cells (Fig. 3a). MitoTracker staining revealed a significantly lower mitochondria mass in GLO1^−/−^ hiPS cells compared with that in WT cells (Fig. 3b). We performed mitochondrial structural analysis using transmission electron microscopy (TEM) to further demonstrate mitochondrial differences. The morphology of functionally immature mitochondria is typically characterized by mostly fragmented structures, as observed in the mitochondria of GLO1^−/−^ hiPS cells. Furthermore, the mitochondria in GLO1^−/−^ hiPS cells had less developed cristae, whereas the mitochondria in WT cells had a more active and complex morphology, characterized by elongated structures and a denser matrix (Fig. 3c, d). We analyzed the variations in energy metabolism and mitochondrial function between the two groups. The rate of energy metabolism through glycolysis and OXPHOS, a key metabolic process in human stem cells, was measured using an Agilent Seahorse XF instrument. Compared with WT hiPS cells, GLO1^−/−^ hiPS cells showed significant reductions in both the OCR (Fig. 3e, f) and the ECAR (Fig. 3g, h). The mitochondrial ATP production rate was also significantly lower in GLO1^−/−^ hiPS cells, with an approximately twofold difference compared with the WT cells (Fig. 3i). These findings demonstrate that the absence of GLO1 impairs mitochondrial structure and function in undifferentiated hiPS cells, which may lead to defects in DE differentiation.

### A high dose of CHIR99021 rescues mitochondrial dysfunction in GLO1^−/−^ hiPS cells during DE differentiation

**Fig. 4:**
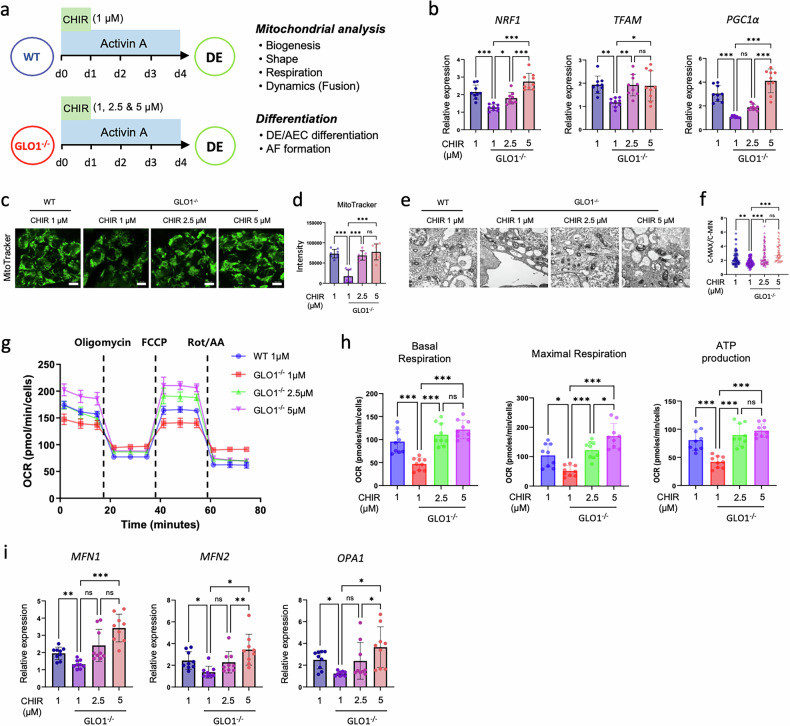
High-dose CHIR99021 restores mitochondrial function in GLO1^−/−^ hiPS cells. **a** Schematic of DE differentiation using a range of doses of CHIR99021 (1, 2.5 and 5 μM) together with Activin A (100 ng/ml) in WT and GLO1^−/−^ hiPS cells. **b** Relative mRNA expression levels of *NRF1*, *TFAM* and *PGC1α* on day 4 were determined by qRT-PCR. **c**, Representative confocal images of mitochondria visualized by MitoTracker Green staining in WT and GLO1^−/−^ hiPS cells. Scale bars, 100 μm. **d**, Mitochondrial masses in WT and GLO1^−/−^ hiPS cells were measured by fluorescence intensity of MitoTracker Green FM staining. **e** Representative TEM images of the mitochondrial structure. **f** Calculated mitochondrial C-MAX/C-MIN values. **g**, The kinetic profile of the OCR was measured in WT and GLO1^−/−^ hiPS cells using the Seahorse XF Real-Time assay. Black lines show times of treatment with oligomycin, FCCP and Rot/AA. **h**, The basal respiration and maximal respiration in WT and GLO1^−/−^ hiPS cells. **i** Relative mRNA expression levels of mitochondrial fusion (*MFN1*, *MFN2* and *OPA1*)-related genes were determined by qRT-PCR. All data are shown as the means ± s.d. *n* = 3 (**b** and **g**–**i**), *n* = 8 (**d**), *n* = 67 (WT 1 μM in **f**), *n* = 80 (GLO1^−/−^ 1 μM in **f**), *n* = 71 (GLO1^−/−^ 2.5 μM in **f**) and *n* = 45 (GLO1^−/−^ 5 μM in **f**). Statistical analyses were performed using a one-way ANOVA and Tukey’s multiple-comparisons test. *P < 0.05, **P < 0.01, ***P < 0.001.

To validate whether mitochondrial dysfunction in GLO1^−/−^ hiPS cells disturbs DE differentiation, we used a pharmacological agent that regulates mitochondrial remodeling. Many studies utilize the glycogen synthase kinase 3 (GSK3) inhibitor, CHIR99021, not only to induce hiPS cells into DE cells but also recently to ameliorate metabolic and inherited diseases by modulating mitochondrial dysfunction^26,27^. Therefore, a range of CHIR99021 doses (0–5 μM) was applied in combination with Activin A to initiate DE differentiation and assess the restoration of DE formation alongside Wnt–β-catenin signaling (Supplementary Fig. 6a,b). In both WT and GLO1^−/−^ hiPS cells, DE differentiation and Wnt–β-catenin signaling increased in a dose-dependent manner with CHIR99021. Notably, in GLO1^−/−^ hiPS cells, concentrations of 2–3 μM or higher elicited differentiation efficiency and signaling activation levels comparable to those observed in WT cells treated with 1 μM CHIR99021. Consequently, we proceeded to examine mitochondrial biogenesis and OXPHOS by treating hiPS cells with 1, 2.5 or 5 μM CHIR99021 during DE differentiation (Fig. 4a). We examined the expression levels of transcription factors related to mitochondrial biogenesis (*NRF1*, *TFAM* and *PGC1α*), and the results revealed the transcriptional recovery of these genes, which had been downregulated in GLO1^−/−^ hiPS cells following initial 24-h treatments of 2.5 and 5 μM CHIR99021, similar to those of the WT control (Fig. 4b). The mass of mitochondria in the GLO1^−/−^ hiPS cells was significantly increased with the 2.5 and 5 μM CHIR99021 treatments compared with 1 μM CHIR99021-treated GLO1^−/−^ hiPS cells (Fig. 4c, d). The mitochondrial morphology of GLO1^−/−^ hiPS cells treated with 2.5 and 5 μM CHIR99021 also showed developed cristae and a denser matrix compared with 1 μM CHIR99021-treated GLO1^−/−^ hiPS cells (Fig. 4e, f). Furthermore, a high dose of CHIR99021 effectively restored reduced OCR values and ATP production in 1 μM CHIR99021-treated GLO1^−/−^ hiPS cells (Fig. 4g, h).

During hiPS cell differentiation, mitochondrial dynamics are crucial for optimal function in energy metabolism. Thus, to further assess whether a high dose of CHIR99021 influences mitochondrial dynamics, the mRNA expression levels of key regulators that coordinate mitochondrial fusion (mitofusin 1 (*MFN1*), *MFN2* and mitochondrial dynamin-like GTPase (*OPA1*)) were determined in WT and GLO1^−/−^ hiPS cells during DE differentiation. The results showed that the reduced mRNA expression levels of these regulators in 1 μM CHIR99021-treated GLO1^−/−^ hiPS cells were significantly upregulated by treatments with 2.5 and 5 μM CHIR99021 (Fig. 4i). These data indicate that mitochondrial dysfunction in GLO1^−/−^ hiPS cells can be pharmaceutically recovered by the upregulation of key factors that regulate mitochondrial biogenesis, respiration and dynamics during DE differentiation.

### High-dose treatment with CHIR99021 rescues the DE and AEC differentiation defects of GLO1^−/−^ hiPS cells

**Fig. 5:**
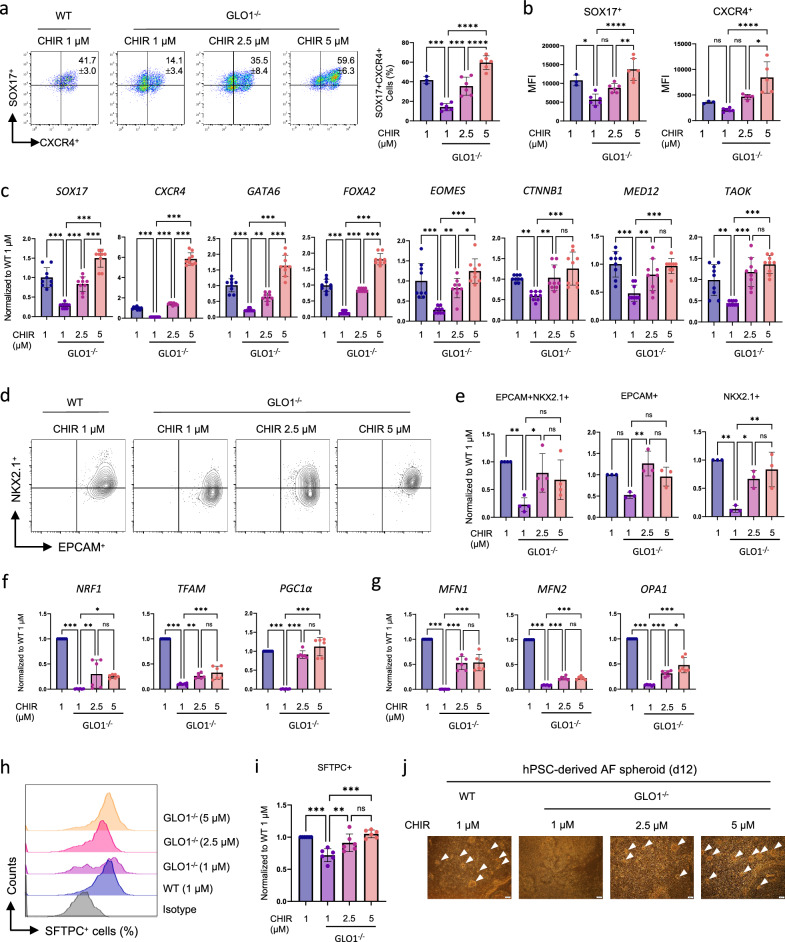
Rescue of the DE and AEC differentiation defects of GLO1^−/−^ hiPS cells by high-dose treatment with CHIR99021. **a** Representative FACS dot plots showing the frequencies of SOX17^+^CXCR4^+^ DE cells (day 4) derived from GLO1^−/−^ hiPS cells treated with CHIR99021 (1, 2.5 and 5 μM). The WT hiPS cells were treated with 1 μM CHIR99021. **b** MFIs of SOX17^+^ and CXCR4^+^ cells in WT and GLO1^−/−^ hiPS cells at day 4 of differentiation. **c** qRT-PCR analysis for DE-positive regulators in WT and GLO1^−/−^ hiPS cells treated with CHIR99021. **d**, Representative FACS dot plots showing the effects of CHIR99021 treatment on the generation of EPCAM^+^NKX2.1^+^ alveolar progenitors from WT and GLO1^−/−^ hiPS cells at day 14 of differentiation. **e**, The graphs show the frequencies of EPCAM^+^NKX2.1^+^, EPCAM+ and NKX2.1 subpopulations. **f**, **g**, Relative mRNA expression levels of mitochondrial biogenesis- (**f**) and fusion- (**g**) related genes in AECs at day 14 of differentiation derived from WT and GLO1^−/−^ hiPS cells were determined by qRT-PCR. **h**, Representative histograms showing the frequencies of SFTPC+ type 2 AECs at day 21 of differentiation. **i**, Data are normalized to the frequency of the WT control treated with 1 μM CHIR99021. All data are shown as the means ± s.d. *n* = 3. Statistical analyses were performed using a one-way ANOVA and Tukey’s multiple-comparisons test. *P < 0.05, **P < 0.01, ***P < 0.001, ****P < 0.0001.

The above results prompted us to investigate whether the recovery of mitochondrial function by CHIR99021 under GLO1 deficiency indeed contributes to DE differentiation and subsequent alveolar development. We administered a range of doses of CHIR99021 (1, 2.5 and 5 μM) on the first day of differentiation for 24 h and then analyzed the cells to assess whether DE–AFE–VAFE–ADAE differentiation was successfully restored at each stage: day 4 (DE), day 12 (AFE), day 14 (VAFE) and day 21 (ADAE). Flow cytometry analysis revealed that treatment with a high dose of CHIR99021 completely restored the defects in DE differentiation observed in GLO1^−/−^ hiPS cells. The frequencies and MFIs of SOX17^+^ and CXCR4^+^ cells were also significantly recovered by 5 µM CHIR99021 treatment in GLO1^−/−^ hiPS cells (GLO1^−/−^ 1 µM versus GLO1^−/−^ 2.5 µM versus GLO1^−/−^ 5 µM, 14.1 ± 3.4% versus 41.7 ± 3.0% versus 59.6 ± 6.3%, *P* < 0.001) (Fig. 5a, b). In addition, the genes that positively regulate DE differentiation were transcriptionally recovered by 2.5 and 5 µM CHIR99021 treatment in GLO1^−/−^ hiPS cells (Fig. 5c).

To further assess whether the recovery effects of CHIR99021 on DE differentiation under GLO1 deficiency positively influence the late stage of alveolar development, the frequencies of both NKX2.1^+^ AEPs and SFTPC^+^ type 2 AECs were measured by flow cytometry. We found that treatment with a high dose of CHIR99021 resulted in higher frequencies of cells expressing EPCAM and NKX2.1 at day 14 of differentiation (Fig. 5d, e). As expected, on day 14 of differentiation, EPCAM^+^NKX2.1^+^ AEPs that regained differentiation capacity following treatment with CHIR99021 exhibited restored mitochondrial biogenesis and dynamics (Fig. 5f, g). The frequency of SFTPC^+^ cells was significantly increased in GLO1^−/−^ hiPS cells treated with a high dose of CHIR99021 at day 21 of differentiation (Fig. 5h, i). Moreover, the defect in AF spheroid formation of GLO1^−/−^ hiPS cells could be rescued by high-dose treatment with CHIR99021 (Fig. 5j). Taken together, our findings demonstrated that mitochondrial dysfunction due to GLO1 deficiency disrupts DE and the alveolar development of hiPS cells, which can be effectively recovered via mitochondrial remodeling by high-dose treatment with CHIR99021.

### GLO1 deficiency negatively regulates β-catenin activity during DE differentiation

**Fig. 6:**
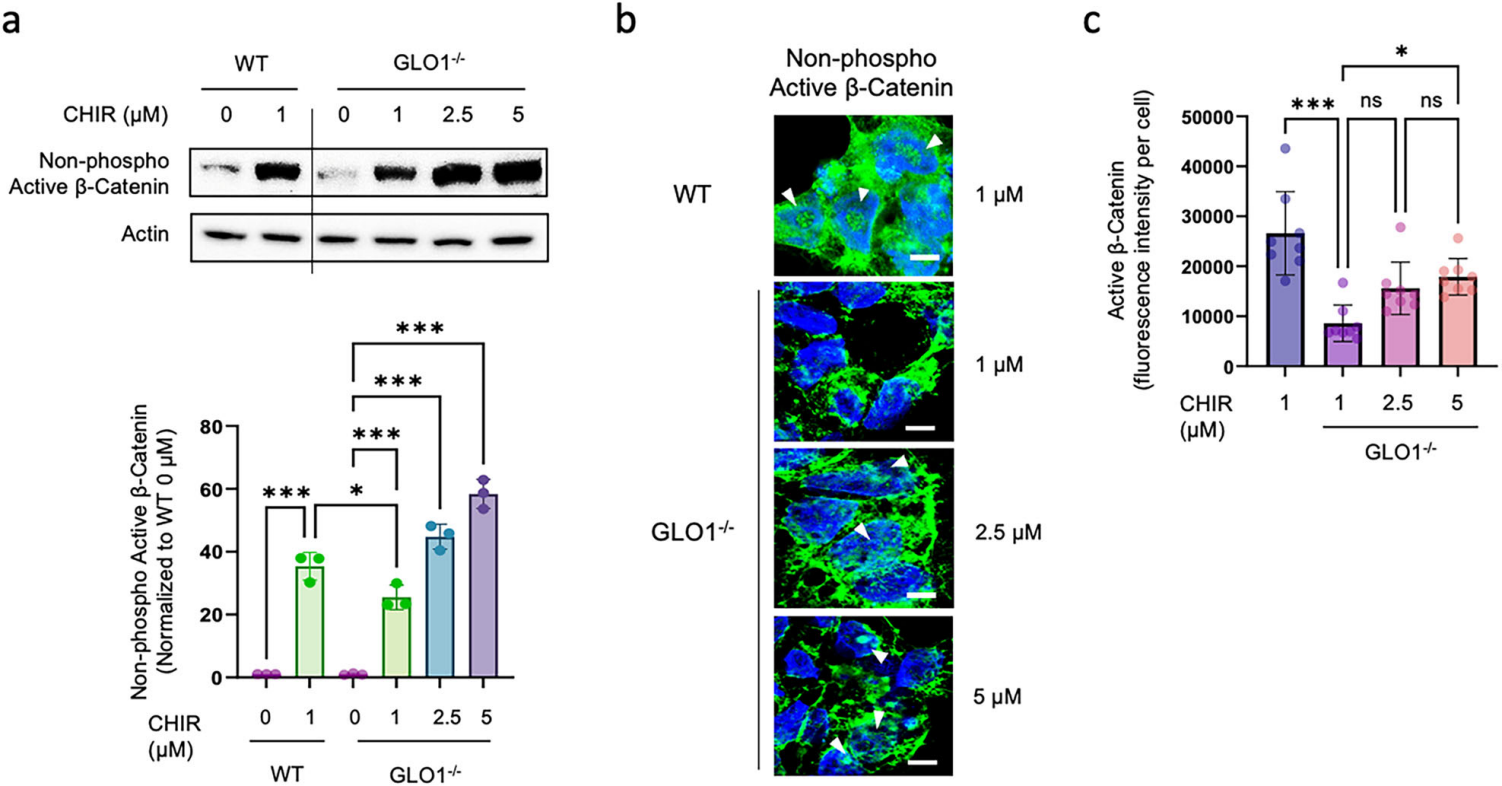
Effects of high-dose CHIR99021 treatment on the expression and nuclear translocation of β-catenin in GLO1^−/−^ hiPS cells during DE induction. **a** Non-phospho (active) β-catenin protein levels in WT and GLO1^−/−^ hiPS cells were analyzed by western blot analysis after 3 h of DE differentiation with 0, 1, 2.5 and 5 μM CHIR99021 treatment. **b**, Representative fluorescence images showing immunocytochemistry staining of non-phospho (active) β-catenin protein in WT and GLO1^−/−^ hiPS cells after 3 h of DE differentiation with 1, 2.5 and 5 μM CHIR99021. **c**, The bar graph represents the average fluorescence intensity of non-phospho (active) β-catenin in WT and GLO1^−/−^ hiPS cells. Western blot analysis was performed by collecting total protein lysates to compare non-phospho (active) β-catenin expression in response to different concentrations of CHIR99021 (0, 1, 2.5 and 5 μM) in WT and GLO1^−/−^ hiPS cells. All data are shown as the means ± s.d. *n* = 3. Statistical analyses were performed using a one-way ANOVA and Tukey’s multiple-comparisons test. ns not significant, *P < 0.05, **P < 0.01.

CHIR99021 is a critical factor for the efficient differentiation of hiPS cells into DE by enhancing β-catenin expression and nuclear translocation^28^. Thus, we assumed that GLO1 might be involved in regulating the activity of β-catenin during DE differentiation. In contrast to WT control hiPS cells, GLO1^−/−^ hiPS cells failed to enhance β-catenin expression and nuclear translocation by CHIR99021 treatment at a low concentration (1 μM) during DE induction. However, high-dose treatment of CHIR99021 (2.5 and 5 μM) resulted in a pronounced increase in non-phospho (active) β-catenin levels and facilitated nuclear transport in GLO1^−/−^ hiPS cells at day 4 of DE induction (Fig. 6a–c). Furthermore, we found that two other WNT agonists, BIO and WNT3a, effectively rescued DE differentiation of GLO1^−/−^ hiPS cells (Supplementary Fig. 7). These findings indicate that GLO1 deficiency negatively regulates the activity of β-catenin during DE differentiation by suppressing its expression and nuclear translocation.

## Discussion

GLO1 is increasingly recognized for its involvement in regulating mitochondrial homeostasis, which plays vital roles in the proliferation, differentiation and regenerative potential of stem cells. Here, we investigated the role of GLO1 during DE differentiation through CRISPR–Cas9-mediated genome deletion in hiPS cells. Our results showed that GLO1 deficiency disturbed DE differentiation of hiPS cells, which in turn led to defects in AEC differentiation and AO development. In addition, GLO1 deficiency interfered with mitochondrial biogenesis and respiration during the early DE stage. Moreover, the defect in DE differentiation due to dysfunctional mitochondria and β-catenin inactivation could be effectively rescued by treatment with a high dose of CHIR99021. This is the first study to demonstrate a previously unrecognized and essential role of GLO1 in early lineage specification, moving away from its conventional role as a primary enzyme in MG detoxification.

The regenerative capacity of tissue-specific stem cells can be impaired by the formation and accumulation of MG under hyperglycemic conditions. Thus, the elevation of GLO1 expression or activity could be a therapeutic option for hyperglycemia-induced tissue injury. For example, the overexpression of GLO1 reverses the defective function of diabetic adipose- and bone marrow-derived mesenchymal stem cells for wound healing in diabetic foot ulcers and neovascularization of diabetic limb ischemia^29,30^. The impaired angiogenic and vasculogenic potential of human dental pulp stem cells by MG-dependent glycative stress can be restored by the induction of GLO1 activity^31^. In addition, reduced regenerative and proangiogenic abilities of cardiac stem cells to repair injured myocardium under hyperglycemic conditions can be restored by increasing the expression of GLO1^32^. These data prompted us to measure the MG levels in undifferentiated GLO1^−/−^ hiPS cells and DE cells, and the results revealed no significant difference between WT and GLO1^−/−^ hiPS cells. These results indicate that defects in the DE differentiation of GLO1^−/−^ hiPS cells are primarily due to mitochondrial dysfunction and inactivation of the β-catenin pathway, not an accumulation of cytotoxic MG. More interestingly, this finding also implies that hiPS cells may have other compensatory systems for metabolizing MG. Recently, D-lactate dehydrogenase has been identified as another enzyme for MG detoxification. D-lactate dehydrogenase catalyzes D-lactate into D-pyruvate, which enters the tricarboxylic acid cycle for ATP production. MG has two functional groups, which act as substrates for oxidoreductase and dehydrogenase enzymes. Thus, various aldo–keto reductases and dehydrogenases have been linked with MG detoxification, in which MG is reduced to acetol or pyruvate using nicotinamide adenine dinucleotide (NADH) or nicotinamide adenine dinucleotide phosphate (NADPH). Therefore, it would be interesting to further explore which of these enzymes is the most effective for MG detoxification and how these enzymes regulate pluripotency and the early lineage specification of hiPS cells.

Our study identifying the pivotal role of GLO1 in normal mitochondrial homeostasis was limited to DE differentiation, but several studies have reported that mitochondrial homeostasis also regulates early lineage specification toward definitive mesoderm and DE. For instance, during the early stage of hiPS cell differentiation, the transcript levels of paired box 6 (*PAX6*) (ectoderm marker), *Brachyury* and mix paired-like homeobox (*MIXL1*) (mesoderm markers) were upregulated along with a concomitant increase in *PGC1α*, a central inducer of mitochondrial biogenesis. Deletion of TFAM, which is also essential for mitochondrial biogenesis^33^, hinders the multilineage specification of hiPS cells toward all three germ layers^8^. Interestingly, despite lower mitochondrial mass in the early mesodermal stage of hiPS cells, the remaining mitochondria in this stage showed maximal OCR and spare capacity compared with the late maturation stage of cardiomyocytes^34^. These findings strongly suggest that defects in mitochondrial homeostasis due to GLO1 deficiency may also negatively influence early mesodermal and ectodermal lineage specification from hiPS cells. Indeed, we observed significant reductions in *Brachyury*, *MIXL1* and *PAX6* mRNA expression levels and lower efficiencies of hematopoietic and neural differentiation in GLO1^−/−^ hiPS cells compared with the WT control cells (data not shown). Therefore, further studies exploring the mechanisms by which GLO1 regulates early mesoderm and ectoderm specification are crucial for revealing the distinct mitochondrial remodeling that controls the specification of the three germ layers.

In our study, we detected the reduced activity of β-catenin in DE cells of GLO1^−/−^ hiPS cells, which was effectively recovered by increasing the concentration of CHIR99021. This report suggests that GLO1 may serve as a positive regulator of β-catenin activity during early lineage specification in hiPS cells. However, it remains entirely unexplored whether β-catenin activated by CHIR99021 in GLO1^−/−^ hiPS cells could recover mitochondrial dysfunction during DE induction. Although the role of β-catenin in the regulation of mitochondrial homeostasis has not been determined in human and murine pluripotent stem cells, it has been well established that β-catenin plays a critical role in the maintenance of mitochondrial homeostasis to ameliorate pathological development. For instance, kidney function loss and mitochondrial dysfunction were exacerbated in mice with tubule-specific β-catenin deletion by suppression of the protein kinase B (AKT)/p53 and forkhead box O3 (FOXO3)/PGC1α signaling pathways^35^. In mice with hepatocyte-specific deletion of β-catenin, ethanol feeding led to a deterioration in mitochondrial function, which included reductions in OXPHOS and ATP levels^36^. Furthermore, activation of β-catenin signaling by genetic knockdown of Axin-2 enhanced mitochondrial biogenesis and dopaminergic neurogenesis in rat models of Parkinson’s disease^37^. More importantly, Ma et al. showed that CHIR99021 promotes mitochondrial biogenesis via β-catenin signaling during the DE differentiation of human adipose tissue-derived stem cells^38^. All these findings strongly support the possibility that GLO1-mediated β-catenin activation is involved in the maintenance of mitochondrial homeostasis during the early lineage specification of hiPS cells. Therefore, understanding molecular mechanisms underlying the mitochondrial functions regulated by β-catenin that are relevant for DE specification of hiPS cells is of great interest to acquire functional AECs and AOs for regenerative medicine and pharmacological testing.

In conclusion, our study uncovered an unconventional regulatory role of GLO1 that contributes to early DE differentiation of hiPS cells through mitochondrial homeostasis. These findings set the stage for expanding the novel role of GLO1 to other lineages, including ectoderm and mesoderm, helping us to identify associated molecules and signaling pathways responsible for early lineage specification. Furthermore, the enhancement of GLO1 activity through genetic or chemical approaches may improve the induction efficiency of hiPS cells so that a sufficient quantity of functional AECs can be produced to provide a better AO model for cell therapy and drug testing for chronic respiratory diseases such as pulmonary fibrosis and chronic obstructive pulmonary disease.

## Supplementary information

Supplementary Information
