# Supplementary material for: Mitochondrial dysfunction by glyoxalase 1 deficiency disrupts definitive endoderm and alveolar development of human pluripotent stem cells
Source: Exp Mol Med. 2025 Sep 1;57(9):1940–50. doi: 10.1038/s12276-025-01524-y (PMC12508193; doi:10.1038/s12276-025-01524-y)
Supplement: Supplementary file 1 — Supplementary Information [file 12276_2025_1524_MOESM1_ESM.pdf]

Supplementary Information for

Mitochondrial dysfunction by glyoxalase 1 deficiency disrupts definitive endoderm and alveolar development of human pluripotent stem cells

Suji Jeong[1#], Hyebin Koh[2,3#], Minje Kang[1], Ji-Young Kim[1], Roya Rasaei[1], Woo Jin Kim[1], Seon-Sook Han[1], In Sun Hong[4], Se-Ran Yang[5], Jong-Hee Lee[2,3]*, Seok-Ho Hong[1.6]*

[1] *Department of Internal Medicine, School of Medicine, Kangwon National University, Chuncheon, Republic of Korea*
[2] *National Primate Research Center (NPRC), Korea Research Institute of Bioscience and Biotechnology (KRIBB), Cheongju, Republic of Korea*
[3] *Department of Advance Bioconvergence, KRIBB School of Bioscience, University of Science and Technology (UST), Daejeon, Republic of Korea*
[4] *Department of Biochemistry, School of Medicine, Gachon University, Incheon, Republic of Korea*
[5] *Department of Thoracic and Cardiovascular Surgery, School of Medicine, Kangwon National University, Chuncheon, Republic of Korea*
[6] *KW-Bio, Chuncheon, Republic of Korea*

Supplementary material contains

Supplementary Fig. 1 Temporal expression of GLO1 during hPSC-derived AEP differentiation

Supplementary Fig. 2 Generation and characterization of GLO1$^{-/-}$ hPSCs using CRISPR/Cas9-mediated genome deletion

Supplementary Fig. 3 Temporal expression of CXCR4 during DE differentiation in WT and GLO1$^{-/-}$ hiPSCs

Supplementary Fig. 4 Comparison of necrotic percentages between WT and GLO1$^{-/-}$ hiPSCs

Supplementary Fig. 5 Expression of various lung cell-type markers in AF spheroid-lung organoids (LOs) from WT hiPSCs

Supplementary Fig. 6 CHIR99021 dose-dependent DE differentiation and WNT/β-catenin signaling activation

Supplementary Fig. 7 DE differentiation efficiency of WT and GLO1$^{-/-}$ hiPSCs in response to WNT agonists (BIO and WNT3a)

Supplementary Table 1. Primer sequences for qRT-PCR

Supplementary Table 2. List of antibodies for FACS analysis

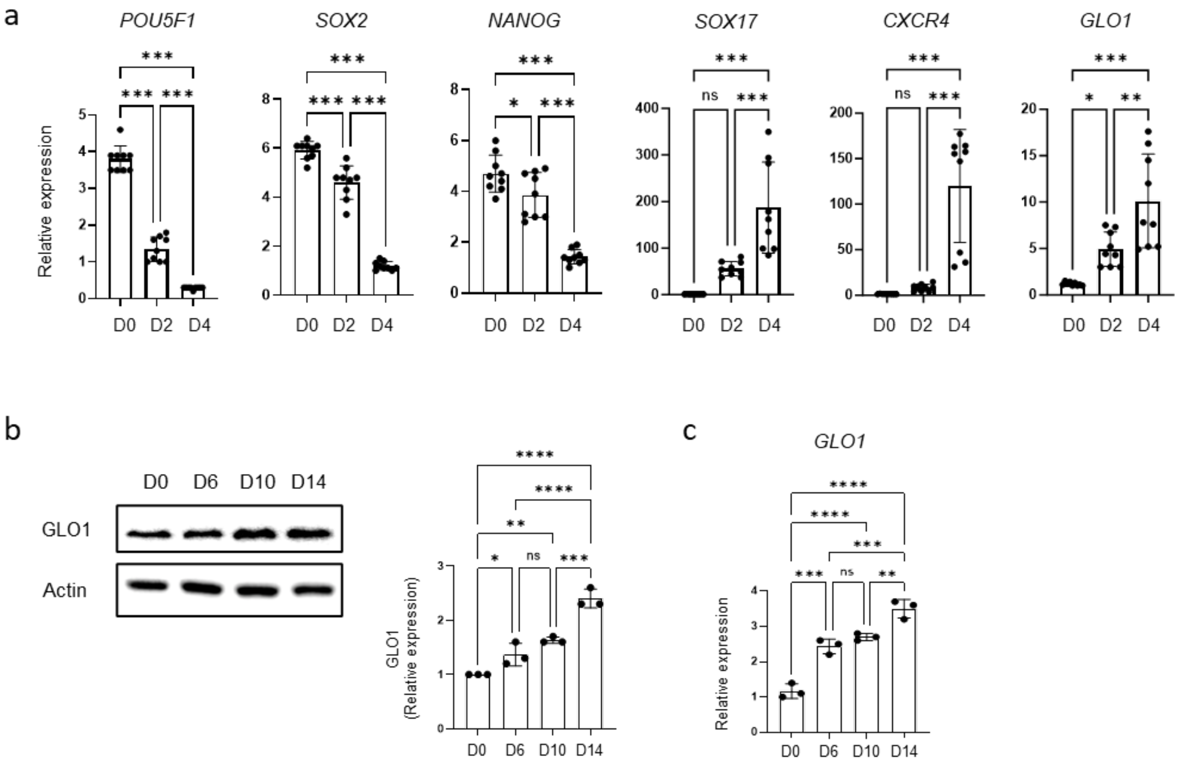

Supplementary Fig. 1 Temporal expression of GLO1 during hiPSCs-derived AEP differentiation

a qRT-PCR analysis of pluripotency (*POU5F1*, *SOX2 and NANOG*), DE-specific (*SOX17 and CXCR4*) and *GLO1* genes in WT hPSCs at DE day 4. Data are normalized to the mRNA level of day 0. **b** Western blot analysis of GLO1 protein expression in WT hPSCs at various differentiation stages (D0, D6, D10 and D14). **c** qRT-PCR analysis of *GLO1* gene expression in WT hiPSCs at various differentiation stages **(**D0, D6, D10 and D14**).** All data are shown as mean ± SD. n=3. Statistical analyses were performed using an unpaired student's t-test. *p<0.05, **p<0.01 and ***p<0.001.

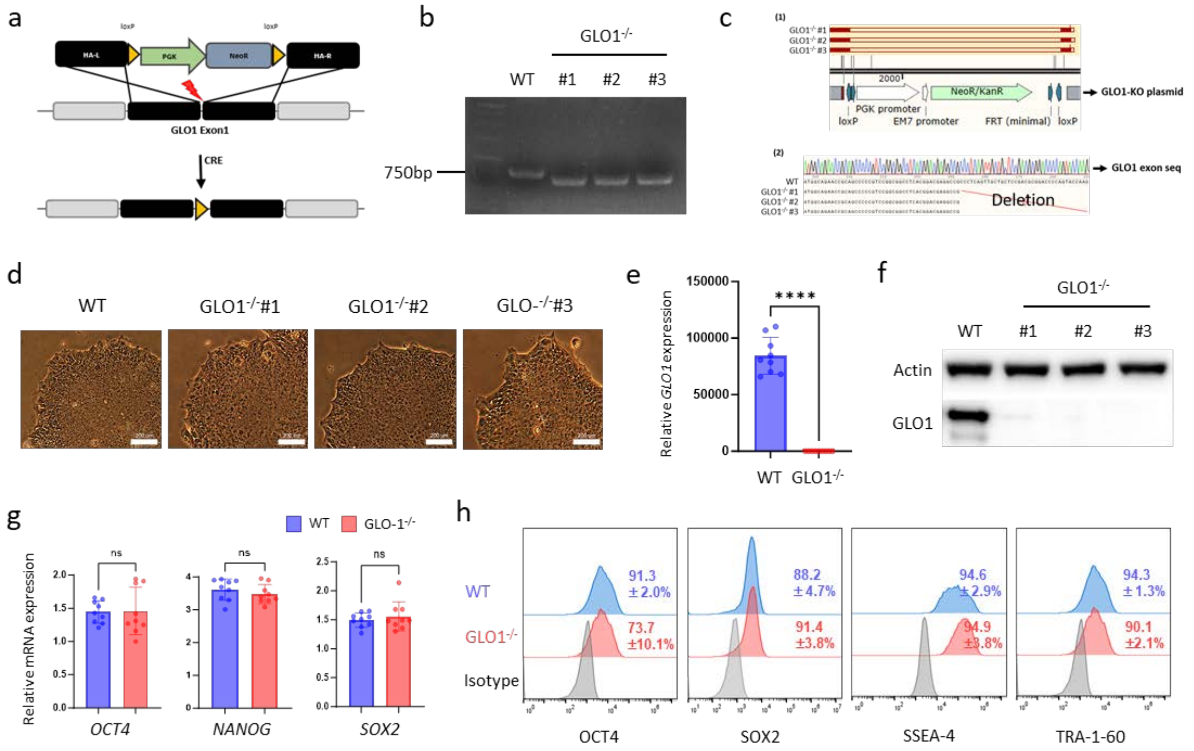

Supplementary Fig. 2 Generation and characterization of GLO1$^{-/-}$ hiPSCs using CRISPR/Cas9-mediated genome deletion

a Schematic representation of the *GLO1* gene editing. **b** Analysis of *GLO1* genome editing results through gDNA PCR. **c** Sanger sequencing analysis of GLO1$^{-/-}$ hiPSCs after Cre recombinase treatment, confirming complete knockout of the PGK-NeoR selection cassette and the GLO1 exon. **d** Representative bright-field images of undifferentiated WT and GLO1$^{-/-}$ hiPSCs. Bars, 200 μm. **e** Relative mRNA expression levels of *GLO1* gene in undifferentiated WT and GLO1$^{-/-}$ hiPSCs. **f** Western blot analysis of GLO1 protein expression in undifferentiated WT and GLO1$^{-/-}$ hiPSCs. Representative blots are shown for WT and three independent GLO1$^{-/-}$ clones (#1, #2, #3). **g** Relative mRNA expression levels of pluripotency markers (*OCT4*, *NANOG* and *SOX2*) in undifferentiated WT and GLO1$^{-/-}$ hiPSCs. **h** Flow cytometric analysis of pluripotency markers (OCT4, SOX2, SSEA-4 and TRA-1-60) in undifferentiated WT and GLO1$^{-/-}$ hiPSCs. All data are shown as mean ± SD. n=3. Statistical analyses were performed using an unpaired student's t-test. ns = not significant and ***p<0.001.

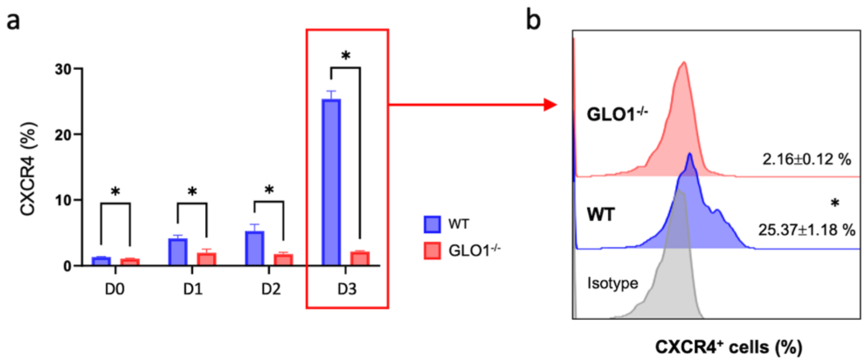

Supplementary Fig. 3 Temporal expression of CXCR4 during DE differentiation in WT and GLO1$^{-/-}$ hiPSCs

a Flow cytometric analysis to determine temporal induction efficiency between WT and GLO1$^{-/-}$ hiPSCs. **b** Flow cytometric analysis of the DE differentiation between WT and GLO1$^{-/-}$ hiPSCs on day 3 of differentiation. CXCR4^{+} cells represent DE cells. All data are shown as mean ± SD. n=3. Statistical analyses were performed using an unpaired Student's *t*-test. *p<0.05.

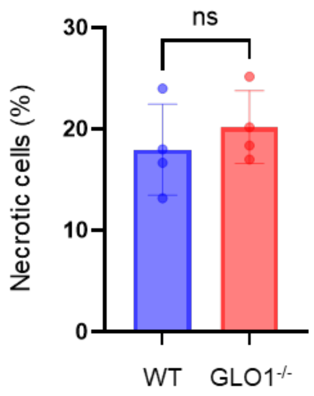

Supplementary Fig. 4 Comparison of necrotic percentages between WT and GLO1$^{-/-}$ hiPSCs

Flow cytometric analysis of WT and GLO1$^{-/-}$ hiPSCs using Annexin V and Propidium Iodide (PI) staining. Necrotic cells (%) were calculated from the Annexin V^{-}PI^{+} population and shown in the graph. All data are shown as mean ± SD. n=3. Statistical analyses were performed using an unpaired student's t-test. ns = not significant

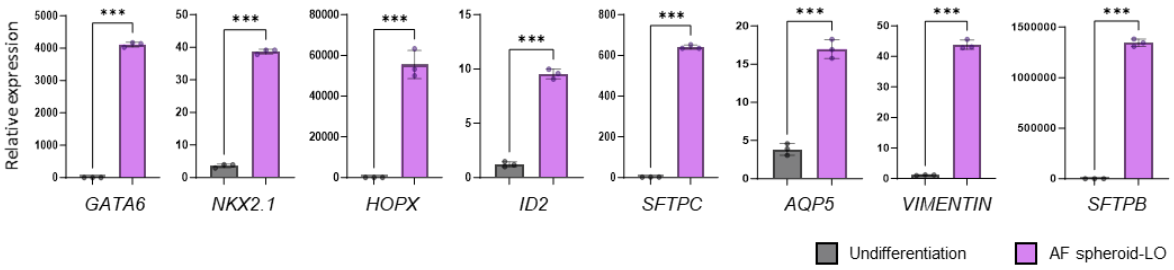

Supplementary Fig. 5 Expression of various lung cell-type markers in AF spheroid-lung organoids (LOs) from WT hiPSCs

qRT-PCR analysis of gene expression for specific lung cell markers in AF spheroid-LOs differentiated from WT hiPSCs. Markers for AEPs (*GATA6* and *NKX2.1*), type 1 AECs (*HOPX* and *AQP5*), type 2 AECs (*SFTPC* and *SFTPB*), distal tip lung progenitor cells (*ID2*) and mesenchymal stromal cells (*Vimentin*) were analyzed. All data are shown as mean ± SD. n=3. Statistical analyses were performed using an unpaired student's t-test. *p<0.05, **p<0.01 and ***p<0.001.

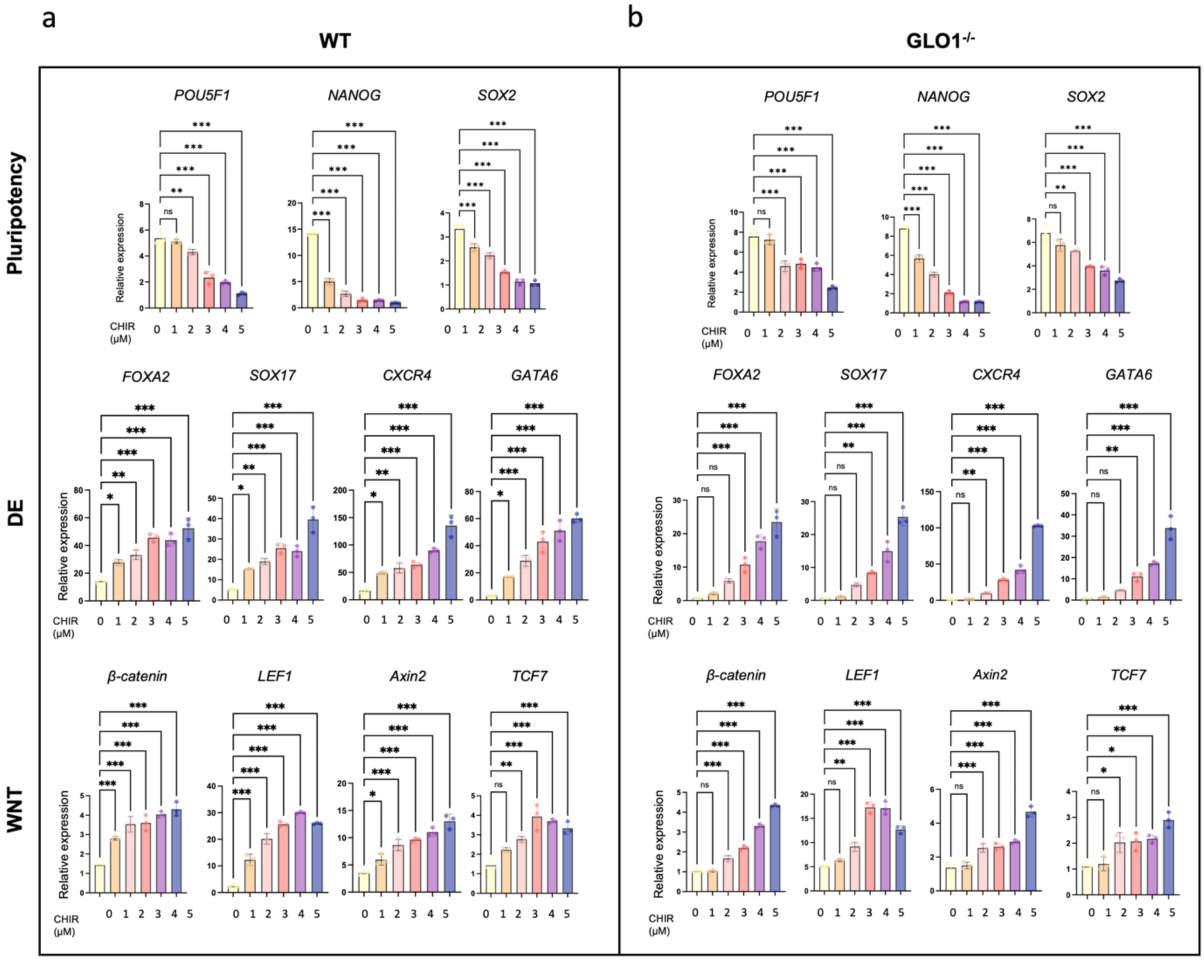

Supplementary Fig. 6 CHIR99021 dose-dependent DE differentiation and WNT/β-catenin signaling activation

a, b qRT-PCR analysis of DE-specific genes (*FOXA2*, *SOX17*, *CXCR4* and *GATA6*) and Wnt/β-catenin signaling-related genes in WT (**a**) and GLO1$^{-/-}$ (**b**) hiPSCs treated with CHIR99021 (0, 1, 2, 3, 4 and 5 μM). All data are shown as mean ± SD. n=3. Statistical analyses were performed using an unpaired Student's *t*-test. *p<0.05, **p<0.01 and ***p<0.001.

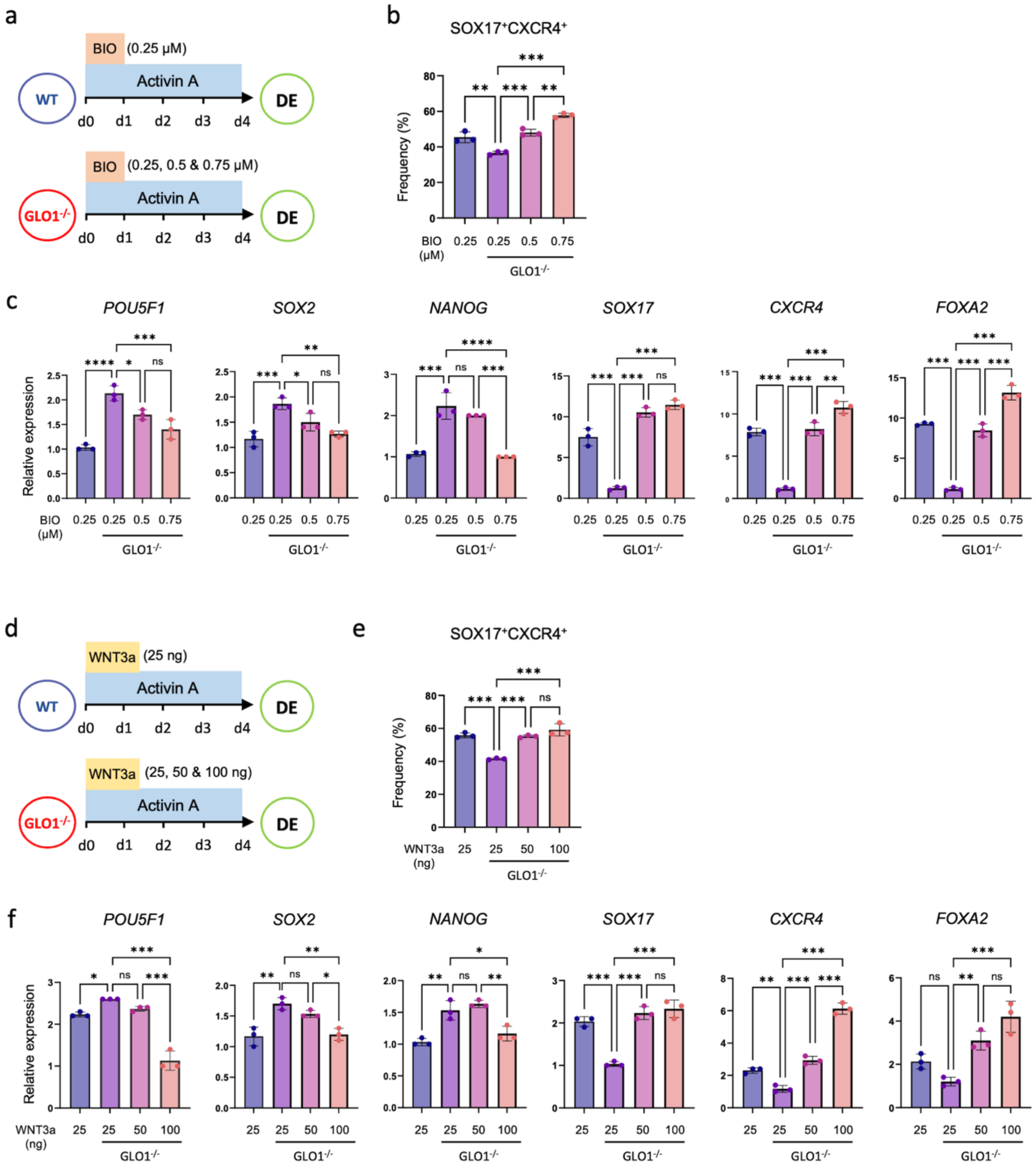

Supplementary Fig. 7 DE differentiation efficiency of WT and GLO1$^{-/-}$ hiPSCs in response to different concentrations of other WNT agonists (BIO and WNT3a)

a Schematic representation of the DE differentiation protocol treatment of BIO. **b** Flow cytometric analysis of day 4 DE cells **(**SOX17^{+}CXCR4^{+}**)** in WT and GLO1$^{-/-}$ hiPSCs treated with BIO (0.25, 0.5 and 0.75 µM). **c** qRT-PCR analysis of pluripotency (*POU5F1***,** *SOX2* and *NANOG*) and DE-specific (*SOX17***,** *CXCR4* and *FOXA2*) genes in WT and GLO1$^{-/-}$ hiPSCs at day 4 under the same BIO treatment conditions. **d** Schematic representation of the DE differentiation protocol treatment of WNT3a. **e** Flow cytometric analysis of day 4 DE cells **(**SOX17^{+}CXCR4^{+}**)** in WT and GLO1$^{-/-}$ hiPSCs treated with WNT3a (25, 50 and 100 ng). **f** qRT-PCR analysis of pluripotency (*POU5F1***,** *SOX2* and *NANOG*) and DE-specific (*SOX17***,** *CXCR4* and *FOXA2*) genes in WT and GLO1$^{-/-}$ hiPSCs at day 4 under the same WNT3a

treatment conditions. All data are shown as mean ± SD. n = 3. Statistical analyses were performed using an unpaired Student's *t*-test. *$p<0.05$, **$p<0.01$ and ***$p<0.001$.

Supplementary Table 1. Human primer sequences used for quantitative PCR

Genes		Sequence 5' to 3'	Product size (bp)
GAPDH	F	TGCACCACCAACTGCTTAGC	87
	R	GGCATGGACTGTGGTCATGAG	
NANOG	F	CAAAGGCAAACAACCCACTT	158
	R	TCTGCTGGAGGCTGAGGTAT	
POU5F1	F	TCGAGAACCGAGTGAGAGG	125
	R	GAACCACACTCGGACCACA	
SOX2	F	GCACATGAAGGAGCACCCGGATTA	86
	R	CGGGCAGCGTGTACTTATCCTTCTT	
GATA6	F	ACTTGAGCTCGCTGTTCTCG	107
	R	CAGCAAAAATACTTCCCCCA	
SOX17	F	CGCTTTCATGGTGTGGGCTAAGGACG	186
	R	TAGTTGGGGTGGTCCTGCATGTGCTG	
CXCR4	F	CACCGCATCTGGAGAACCA	78
	R	GCCCATTTCCTCGGTGTAGTT	
FOXA2	F	TCGCTCTCCTTCAACGACTGTTTCC	107
	R	TTCTCGAACATGTTGCCCGAGTCAG	
PTPN14	F	TGGAAAGCACAGGGCAAGAA	101
	R	TTGCTGGCTCTTGCTGAGAA	
CTNNB1	F	TTGAAGGTTGTACCGGAGCC	100
	R	AGCAGCTGCACAAACAATGG	
SMAD2	F	TCGAAAAGGATTGCCACAT	136
	R	AGGGTTTACACATACTTCATCC	
TAOK	F	CCTAGAGCTGGAATGCCGTC	120
	R	GCATGCTCTAAGTCCTTCTGAGT	
MED12	F	CCAGAACCAGCCACTACCTG	107
	R	GCAGCACTCCAGGGTAAGTT	
MIXL-1	F	GGATCCAGGTATGGTTCCAG	219
	R	GGAGCACAGTGGTTGAGGAT	
TGF	F	TGTGTCATTGGGCGCCTG	120
	R	GCCTTGATGCCGGGCAAA	
ACVR1B	F	AGCACCTCGTGTGTGTTCTT	117
	R	CACAGCAGAGCACAAATGTCA	
NKX2.1	F	AGCACACGACTCCGTTCTCA	75
	R	CCTCCATGCCCACTTTCTTG	
EPCAM	F	AGAACCTACTGGATCATCATTGAACTAA	101
	R	CGCGTTGTGATCTCCTTCTG	
NRF1	F	GGCAACAGTAGCCACATTGGCT	143
	R	GTCGTCTGGATGGTCATCTCAC	
TFAM	F	GGCAAGTTGTCCAAAGAAACC	86
	R	GCATCTGGGTTCTGAGCTTTA	
PGC1	F	CGGCTTCTTTGGCTATGACA	104
	R	TGCGCATAGACGAAGTTGGT	
MFN1	F	GAGGTGCTATCTCGGAGACAC	113
	R	GCCAATCCCACTAGGGAGAAC	

MFN2	F	CACATGGAGCGTTGTACCAG	103
	R	TTGAGCACCTCCTTAGCAGAC	
OPA1	F	TGTGAGGTCTGCCAGTCTTTA	140
	R	TGTCCTTAATTGGGGTCGTTG	
HOPX	F	GCCTTTCCGAGGAGGAGAC	97
	R	TCTGTGACGGATCTGCACTC	
ID2	F	GACAGCAAAGCACTGTGTGG	102
	R	TCAGCACTTAAAAGATTCCGTG	
SFTPB	F	GCCATACCACAGGCAATGCT	80
	R	TGCTGCTCCACAAATTGCTT	
SFTPC	F	CCTTCTTATCGTGGTGGTGGT	96
	R	TCTCCGTGTGTTTCTGGCTCAT	
AQP5	F	ACTGGGTTTTCTGGGTAGGG	172
	R	ATGGTCTTCTTCCGCTCTTC	
VIMENTIN	F	CCAGGCAAAGCAGGAGTC	212
	R	CGAAGGTGACGAGCCATT	
GLO1	F	ATGCGACCCAGAGTTACCAC	152
	R	CCAGGCCTTTCATTTTACCA	

Supplementary Table 2. List of antibodies for FACS analysis

Antibodies	Source	Identifier
CXCR4	Biolegned	306510
SOX17	BD Pharmingen	562205
NKX2.1	Abcam	ab76013
EPCAM	Biolegned	324204
SFTPC	Abcam	ab40879
OCT4	BD Pharmingen	560217
NANOG	BD Pharmingen	560483
SSEA-4	BD Pharmingen	560308
TRA-1-60	BD Pharmingen	560173
7-amino actinomycin D	BD Pharmingen	559925
Mouse IgG1, κ Isotype Control	BD Pharmingen	555909
PE Mouse IgG1, κ Isotype Control	BD Pharmingen	555749
APC Mouse IgG1, κ Isotype Control	BD Pharmingen	555751
